# Effectiveness of Nutritional Therapy Versus Alpha-Glucosidase Inhibitors in Postprandial Glycemia Control in an Indian Subpopulation of Patients With Type 2 Diabetes: A Prospective Crossover Study

**DOI:** 10.7759/cureus.106557

**Published:** 2026-04-06

**Authors:** Praveen Rao Shambhukari, Anudeep Gaddam, Shivaraj S Hanchinal

**Affiliations:** 1 Endocrinology, Konaseema Institute of Medical Sciences & Research Foundation, Amalapuram, IND; 2 Endocrinology, Diabetes and Metabolism, Narayana Medical College, Nellore, Nellore, IND; 3 Endocrinology, Diabetes and Metabolism, Kasturba Medical College, Manipal, Manipal, IND; 4 Department of General Medicine, ESIC (Employees' State Insurance Corporation) Medical College and Hospital, Kalaburagi, IND

**Keywords:** alpha-glucosidase inhibitors, medical nutritional therapy, postprandial glycemia, safety and efficacy, type 2 diabetes mellitus

## Abstract

Background: Type 2 diabetes mellitus (T2DM) poses a significant global health challenge, impacting millions of people worldwide. While many studies focus on HbA1c levels, there remains a need to evaluate glycemic variability and postprandial glucose (PPG) excursions. Therefore, this study was conducted to assess and compare the safety and effectiveness of nutritional therapy and alpha-glucosidase inhibitors in achieving rapid PPG stabilization in an Indian subpopulation of patients with T2DM.

Methods: This short-term open-label study included T2DM patients uncontrolled on metformin and sulfonylurea. The study enrolled 60 participants (40 males, 20 females) with a mean age of 54.06 ± 6.04 years, with the majority being 30-60 years of age (85%). All patients were given intermittent medical nutritional therapy (low glycemic index (LGI) diet) and alpha-glucosidase inhibitors (AGIs; acarbose, voglibose) to manage postprandial glycemia. A rotational design ensured that each group experienced all interventions in varying sequences for a comprehensive analysis of their effects. Glycemic responses were assessed using finger-stick capillary glucose measurements and continuous glucose monitoring (CGM) to evaluate postprandial and 24-hour glycemic variability.

Results: Postprandial mean glucose levels were significantly reduced with all interventions (p<0.05), with the highest reduction observed with acarbose. Between AGIs, the adverse effects were similar between medications. The 24-hour mean glucose levels, mean amplitude of glycemic excursion, and area under the curve for 24-hour glycemic fluctuations were significantly lower with interventions, especially acarbose.

Conclusion: We conclude that AGIs and LGI diets effectively reduce postprandial glucose, especially in Indian T2DM patients consuming high-carbohydrate diets with a high glycemic load.

## Introduction

Type 2 diabetes mellitus (T2DM) is a metabolic disorder affecting millions across the world. India has a significant burden of diabetes mellitus (~11.4%) affecting ~101 million people [1]. The majority of the studies conducted focus on glycated hemoglobin (HbA1c) as an endpoint to conclude the effectiveness of treatment. However, HbA1c does not account for glycemic variability [2,3]. The postprandial glycemic excursion is observed in the majority of Indians with T2DM due to a diet rich in simple carbohydrates. Unlike Western countries, postprandial glycemia significantly contributes to overall HbA1c levels across its range; hence, it is crucial to reduce postprandial hyperglycemia to achieve the target HbA1c in most Indians. More importantly, postprandial glycemic excursions have been independently associated with increased risk of cardiovascular events and mortality. Hence, reducing the postprandial glycemic excursion is crucial for Indians with T2DM.

Alpha-glucosidase inhibitors (AGIs) are a class of oral anti-diabetic agents that assist in reducing postprandial blood glucose levels by reducing or delaying carbohydrate digestion and absorption. This is achieved by competitive enzyme inhibition without causing any proactive stimulation of insulin release. In India, AGIs such as acarbose [4] and voglibose [5] have been extensively used as the first-line and second-line therapies in treating patients with T2DM. However, they are associated with various gastrointestinal side effects, including flatulence, abdominal distension, diarrhea, dyspepsia, and nausea [6,7]. Previous studies have explored the efficacy of acarbose and voglibose individually [4,8]. Knowledge of the relative efficacy of these drugs is essential for effective decision-making during clinical practice, in developing guidelines, and in drafting public health policies. Studies exploring the relative efficacies between acarbose and voglibose have been conducted in other countries such as Thailand and Japan [9,10].

Evidence from prospective epidemiological research and clinical trials has converged in recent decades to support the role of particular nutrients, foods, and dietary patterns in preventing and treating T2DM. The diet high in nutritious carbohydrates, vegetables, fruits, lentils, and nuts, in addition to restricted alcohol use, processed meats, refined grains, and sugary drinks, has been shown to cut the risk of T2DM while enhancing glycemic control in diabetes mellitus patients [11]. The glycemic index (GI) compares an individual's blood glucose response to a specific test diet to a reference food (glucose or white bread) with identical carb amounts (typically 50 grams) [12-16]. GI was initially developed in diabetic patients to anticipate postprandial rises in blood glucose concentration [12-14]. Low-GI diets consist of more fiber and have been shown to enhance the overall glycemic profile when used as an intervention for T2DM patients, resulting in lower HbA1c [12].

Although both nutritional therapy and AGIs are established approaches for managing postprandial glycemia, their comparative effectiveness within the context of Indian dietary patterns remains unclear. This study was designed to evaluate and compare the safety and rapidity of PPG stabilization achieved with nutritional therapy versus AGIs in patients with type 2 diabetes in India.

## Materials and methods

Methodology

This short-term, open-label study involved patients with T2DM who were on a stable regimen of metformin and/or sulfonylurea, with no adjustments to their medication having been made in the three months prior to the study and presenting with a PPG level >180 mg/dl and HbA1c >8%. Exclusion criteria included type 1 diabetes mellitus, known hypersensitivity to acarbose and voglibose, significant gastrointestinal diseases, specific cardiovascular conditions, serum creatinine level >1.5 mg/dl, elevated alanine transaminase (ALT) or aspartate transaminase (AST) levels three times or more than the normal, pregnancy and lactation, and any condition that might hinder completion of the study or pose significant risks to the participants. Patients receiving insulin therapy or additional oral antidiabetic agents were excluded to minimize potential confounding effects.

The study was conducted at the Endocrinology outpatient department of Narayana Medical College and Hospital, India, between July 2019 and December 2020. Written informed consent was obtained from all participants prior to enrolment. The study protocol was approved by the Institutional Ethics Committee (on February 22, 2019) and was conducted in accordance with the Declaration of Helsinki and its subsequent amendments.

Study procedure

Before initiation of the crossover interventions, all participants underwent a two-week run-in period during which background antidiabetic therapy and dietary habits were stabilized. This run-in phase was intended to harmonize baseline glycemic parameters and minimize inter-individual variability before crossover exposure.

The subsequent crossover phase was designed to assess acute, meal-specific postprandial glycemic responses, and therefore, no formal washout period was included between intervention days, given the short duration of action and minimal carryover effects of the interventions studied.

During the intervention phase, participants received four predefined interventions administered over four consecutive days. Alpha-glucosidase inhibitors (acarbose and voglibose) were administered as add-on therapy to the existing background antidiabetic treatment in order to evaluate their effect on postprandial glycemic control. These included acarbose 50 mg administered with all three major meals, voglibose 0.3 mg administered with all three major meals, a prescribed low-GI diet provided for all three major meals, and a regular diet without dietary modification. Each participant was exposed to all interventions using a rotational crossover design, ensuring that all treatment conditions were experienced in varying sequences (Table 1).

**Table 1:** Grouping of patients based on interventional strategies GI: glycemic index

	Group A	Group B	Group C	Group D
Day 1	Acarbose	Voglibose	Low-GI diet	Regular diet
Day 2	Regular diet	Low-GI diet	Acarbose	Voglibose
Day 3	Voglibose	Acarbose	Regular diet	Low-GI diet
Day 4	Low-GI diet	Regular diet	Voglibose	Acarbose

The low-GI diet consisted of commonly consumed Indian foods with a GI of ≤55 and was prescribed for breakfast, lunch, and dinner. The diet was isocaloric and provided approximately 50%-55% of total energy from carbohydrates derived from whole grains, millets, and legumes, 15%-20% from protein, and 25%-30% from fat. The diet was characterized by higher dietary fibre content and minimal refined carbohydrates. Portion sizes and meal timings were standardized across participants to ensure consistency. To ensure consistency of dietary exposure during the intervention period, meal composition was standardized using commonly consumed Indian foods. For example, breakfast included two *vadas* prepared from approximately 100 g of *urad dal* served with coconut chutney, while lunch consisted of two chapatis prepared from approximately 150 g of wheat, accompanied by brinjal curry.

Continuous glucose monitoring

All participants underwent continuous glucose monitoring (CGM) using a Medtronic CGM system (Medtronic MiniMed, USA) for a total duration of five days. The sensor was inserted subcutaneously over the anterior abdominal wall and recorded interstitial glucose values at five-minute intervals, generating up to 288 readings per day. These data were used to evaluate postprandial glucose (PPG) responses as well as 24-hour glycemic variability parameters.

Measurement parameters

Meal initiation time, macronutrient composition, and glycemic index of each meal were meticulously recorded. CGM data were used to calculate mean glucose values and area under the curve (AUC) at two, four, and six hours following each meal. Additional CGM-derived metrics included time in range (TIR), time above range (TAR), and time below range (TBR), enabling a comprehensive assessment of postprandial and overall glycemic control.

Study endpoints

The primary endpoint was the CGM-derived, mean two-hour PPG, calculated as the average of two-hour PPG values following breakfast, lunch, and dinner during each intervention condition. Secondary endpoints included four-hour and six-hour PPG levels; the proportion of participants achieving target PPG (<180 mg/dl) at two, four, and six hours; CGM-derived metrics including time in range (TIR), time above range (TAR), and time below range (TBR); 24-hour mean glucose; mean amplitude of glycemic excursion (MAGE); and area under the curve (AUC) for 24-hour glycemic fluctuations. Safety endpoints included the incidence of adverse events, gastrointestinal intolerance, hypoglycemia, laboratory abnormalities, and treatment discontinuation.

Statistical analysis

Data were collected and entered into Microsoft Excel (Microsoft Corporation, Redmond, USA) and analyzed using GraphPad Prism software (GraphPad, San Diego, USA). Continuous variables are presented as means ± standard deviations, while categorical variables are expressed as frequencies and percentages. The primary endpoint was analyzed using repeated-measures analysis of variance (ANOVA) to account for the within-subject crossover design. Secondary outcomes were evaluated using appropriate repeated-measures or categorical statistical tests, as applicable. A two-sided p-value of <0.05 was considered statistically significant for the primary analysis, while secondary analyses were interpreted descriptively without formal adjustment for multiple comparisons.

## Results

A total of 60 participants were enrolled, with 40 male and 20 female participants. The mean age was 54.06 ± 6.04 years, with the majority being 30-60 years of age (85%). The mean duration of diabetes was 13.4 ± 7.1 years, and BMI was 25.7 ± 3.5 kg/m². Prevalent complications included hypertension (70%), dyslipidemia (60%), neuropathy (75%), and nephropathy (30%). Notably, there were no significant gender-based differences in complications (Table 2).

**Table 2:** Demographics and baseline characteristics The table shows baseline demographic characteristics, clinical parameters, and comorbidities of the study population at enrolment. Continuous variables are presented as means ± standard deviations, and categorical variables are expressed as numbers (percentages). No inferential statistical comparisons were performed for baseline characteristics. BMI: body mass index

Parameters	Total population (N = 60)
Age, in years (mean ± SD)	54.06 ± 6.04
BMI, in kg/m^2^ (mean ± SD)	25.7 ± 3.5
Diabetes duration, in years (mean ± SD)	13.4 ± 7.1
Comorbidities and complications, n (%)	
Hypertension	42 (70%)
Hypothyroidism	9 (15%)
Dyslipidemia	36 (60%)
Neuropathy	45 (75%)
Retinopathy	26 (43.3%)
Nephropathy	17 (28.3%)

The CGMS data - TIR, TAR, and TBR - of all patients are shown in Table 3. The proportion of patients achieving target TIR, TAR, and TBR was 1.7%, 5%, and 33.3%, respectively.

**Table 3:** Continuous glucose monitoring system (CGMS) parameters in total population Baseline CGMS parameters in the total study population were derived from five days of monitoring. Time in range (TIR), time above range (TAR), and time below range (TBR) represent the percentage of time glucose values remained within, above, or below predefined glycemic thresholds.

	Total population (N = 60)
TIR (%) (mean ± SD)	45.8 ± 10.3
TAR (%) (mean ± SD)	47.0 ± 11.3
TBR (%) (mean ± SD)	7.3 ± 4.7

**Figure 1:**
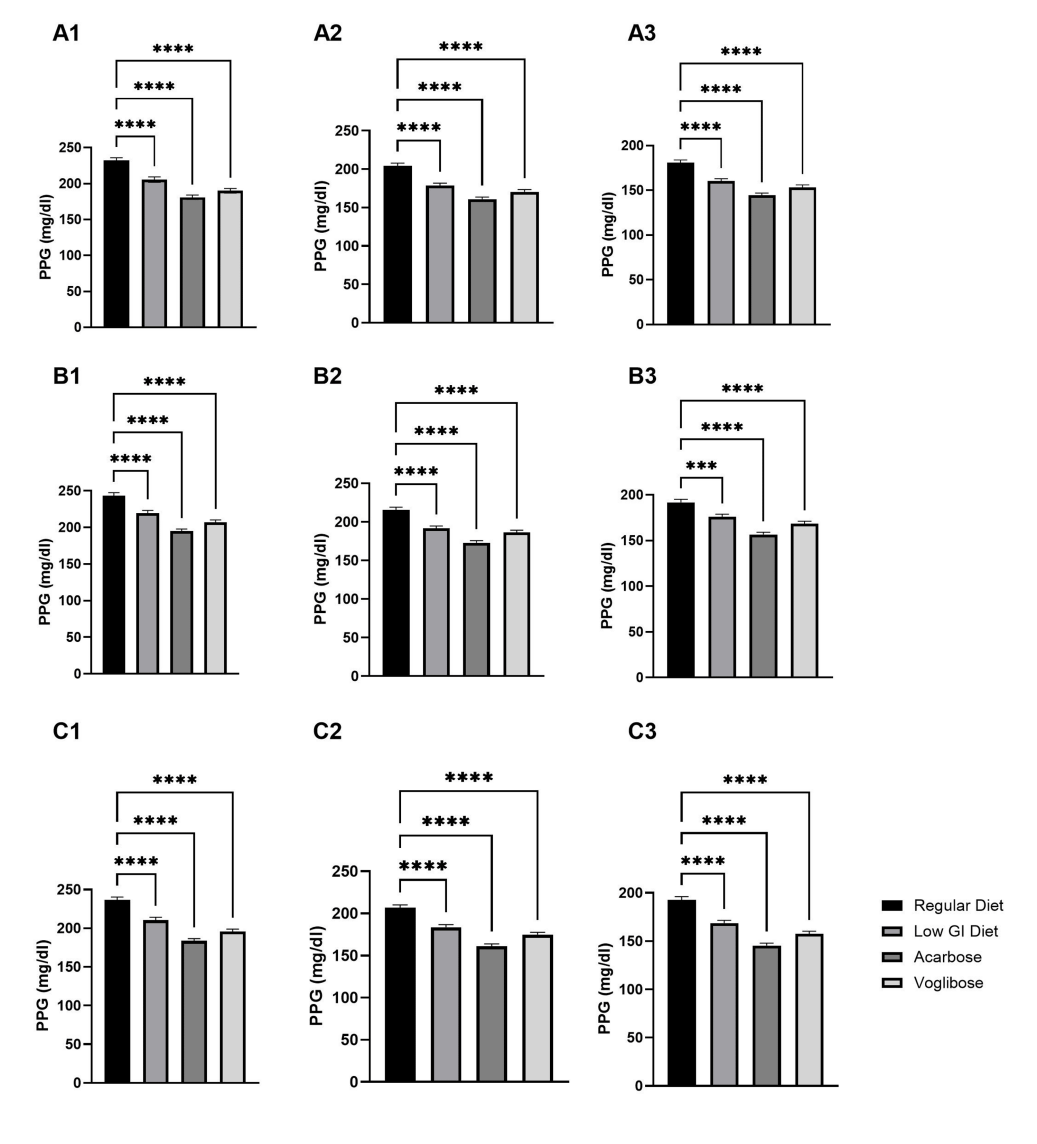
Rapid postprandial glucose (PPG) stabilization with different interventions A1, A2, and A3 represent the two-hour, four-hour, and six-hour PPG levels, respectively, after breakfast, following interventions; B1/B2/B3 represent the two-hour, four-hour, and six-hour PPG levels, respectively, after lunch, following interventions; C1/C2/C3 represent the two-hour, four-hour, and six-hour PPG levels, respectively, after dinner, following interventions. GI: glycemic index

For the primary endpoint, the mean two-hour postprandial glucose across all meals was significantly lower with all active interventions (low-GI diet, acarbose, and voglibose) compared with the regular diet (p<0.05), with the most significant reduction observed with acarbose. Figure 1 presents the comparison of PPG levels for breakfast, lunch, and dinner, respectively, among patients with T2DM under different interventions. Following any intervention (low-GI diet, acarbose, and voglibose), the two-, four-, and six-hour PPG levels were significantly lower than levels in those on a regular diet (Figure 1). Among all interventions, acarbose treatment had the lowest two-, four-, and six-hour PPG levels after meals compared to other interventions.

Table 4 compares the proportion of participants achieving target two-, four-, and six-hour PPG levels (<180 mg/dl). Among the interventions, post-acarbose treatment, a higher proportion of individuals achieved two-, four-, and six-hour glycemic targets (PPG levels <180 mg/dl).

**Table 4:** Population achieving target postprandial glucose (PPG) levels (<180 mg/dl) with different interventions In this table, results for the proportion of participants achieving target PPG levels (<180 mg/dl) at two, four, and six hours following breakfast, lunch, and dinner, under different dietary and pharmacological interventions, are shown. Values are presented as numbers (percentages). Group comparisons were performed using the chi-square test. GI: glycemic index

	Regular diet	Low-GI diet	Acarbose	Voglibose	χ^2^	p-value
Breakfast – 2 hours	1 (1.75%)	14 (24.5%)	31 (51.7%)	19 (31.7%)	40.83	<0.0001
Breakfast – 4 hours	11 (18.3%)	32 (53.22%)	50 (83.3%)	41 (68.3%)	66.97	<0.0001
Breakfast – 6 hours	31 (50.3%)	49 (81.7%)	59 (98.3%)	52 (86.7%)	43.77	<0.0001
Lunch – 2 hours	0 (0)	5 (8.3%)	26 (43.3%)	7 (11.7%)	48.65	<0.0001
Lunch – 4 hours	6 (10%)	20 (33.3%)	37 (61.7%)	21 (35%)	35.31	<0.0001
Lunch – 6 hours	21 (35%)	32 (50.7%)	52 (86.7%)	45 (75%)	40.46	<0.0001
Dinner – 2 hours	0 (0)	8 (13.3%)	26 (43.3%)	16 (26.7%)	37.5	<0.0001
Dinner – 4 hours	9 (15%)	28 (46.7%)	50 (83.3%)	36 (60%)	58.62	<0.0001
Dinner – 6 hours	19 (31.7%)	39 (65%)	59 (98.3%)	52 (86.7%)	74.15	<0.0001

In Table 5, a comparison of glycemic variables among the interventions is shown. Significant differences were noted in the 24-hour mean glucose level, MAGE, and AUC for 24-hour glycemic fluctuations (p<0.05).

**Table 5:** Comparison of glycemic variables with different interventions Herein, a comparison of continuous glycemic parameters across regular diet, low glycemic index diet, acarbose, and voglibose interventions is shown. Values are expressed as means ± standard deviations and derived from continuous glucose monitoring data. Comparisons were performed using repeated-measures analysis of variance (ANOVA), with F statistics used to assess overall differences across interventions. MAGE: mean amplitude of glycemic excursion; AUC: area under the curve

Variables	Regular diet	Low glycemic index diet	Acarbose	Voglibose	F-value	p-value
24-h mean glucose level (mg/dl)	212 ± 27	188 ± 25	167 ± 20	178 ± 21	40.26	<0.0001
MAGE (mg/dl)	104 ± 38	82 ± 34	68 ± 29	73 ± 31	13.83	<0.0001
AUC for 24-h glycemic fluctuations (mg/dl)	4130± 1321	1291 ± 893	584 ± 274	985 ± 322	229.9	<0.0001

Adverse effects of voglibose and acarbose are compared in Table 6. No statistically significant difference was observed in adverse effects between the two medications (p>0.05). Adverse effects, including flatulence, abdominal distension, diarrhea, increased ALT, hypoglycemia, severe adverse events (SAEs), discontinuation due to adverse events, constipation, dyspepsia, upper abdominal pain, and death, were analyzed.

**Table 6:** Comparison of adverse effects between voglibose and acarbose treatment groups Values are presented as numbers (percentages). Group comparisons were performed using the chi-square test or Fisher’s exact test, as appropriate. AE: adverse event; ALT: alanine aminotransferase; SAE: severe adverse event

Adverse effects	Voglibose	Acarbose	z-statistic	p-value
All AEs	29 (48.3%)	37 (61.7%)	-1.47	0.142
Flatulence	27 (45%)	33 (55%)	-1.10	0.273
Abdominal distension/bloating	23 (38.3%)	31 (51.7%)	-1.47	0.142
Diarrhea	6 (10%)	11 (18.3%)	-1.31	0.191
Increased ALT	4 (6.7%)	7 (11.7%)	-0.95	0.343
Hypoglycemia	3 (5%)	7 (11.7%)	-0.95	0.343
SAEs	0 (0%)	0 (0%)	-	-
Discontinuation due to AEs	0 (0%)	0 (0%)	-	-
Constipation	3 (5%)	5 (8.3%)	-0.73	0.464
Dyspepsia	8 (13.3%)	12 (20%)	-0.98	0.327
Upper abdominal pain (mild)	3 (5%)	6 (10%)	-1.04	0.298
Mortality	0 (0%)	0 (0%)	-	-

## Discussion

The current study comparing the rapid PPG stabilization and safety of nutritional therapy (LGI diet) and AGIs (acarbose and voglibose) in Indian T2DM patients observed significant improvement in two-, four-, and six-hour glycemic levels following breakfast, lunch, and dinner. Acarbose treatment resulted in the lowest postprandial glucose levels at two, four, and six hours following breakfast, lunch, and dinner compared to the other interventions. However, only a small proportion of patients achieved target TIR, TAR, and TBR. No statistically significant difference was observed in adverse effects, particularly gastrointestinal side effects, between the two AGIs.

In this study, the majority of participants were male (n = 40; 66.7%) and in the 30-60 years (85%) age group. Three-fourths of the participants were overweight or obese. Urbanization, especially in rural areas, is associated with lifestyle changes that contribute to obesity and metabolic syndrome [17]. Among the study participants, major comorbidities included metabolic disorders such as hypertension (70%) and dyslipidemia (60%). Although these comorbidities were prevalent among the study participants, the present study was not designed to evaluate long-term cardiovascular outcomes. These observations are therefore presented primarily as background clinical context rather than direct study findings [18]. Previous studies have reported that atherosclerotic cardiovascular disease may occur one to two decades earlier in Indian patients with T2DM compared with Western populations [19].

Diabetes complications, including neuropathy (75%), retinopathy (43.3%), and nephropathy (28.3%), were observed in a significant portion of the population (Table 2). Studies have indicated that a diabetes duration of >10 years is strongly associated with an increased risk of microvascular complications [20]. The high prevalence of complications may be attributed to the fact that a majority of the population had a diabetes duration exceeding 10 years.

The two-hour, four-hour, and six-hour PPG levels were significantly lower with any intervention compared to the standard Indian diet. Between AGIs used (acarbose and voglibose), acarbose exhibited a greater reduction in PPG levels at two, four, and six hours after breakfast, lunch, and dinner (Figure 1), and a higher proportion achieved target PPG levels (Table 3). However, the incidence of adverse events was similar between the groups. The results of previous head-to-head comparison studies between acarbose and voglibose in the Indian T2DM population do not align consistently, with some studies showing higher effectiveness with acarbose [21] and some with voglibose [22,23]. Therefore, the choice between acarbose and voglibose should be tailored to individual patient needs, considering factors such as effectiveness and tolerability. Further research is needed to clarify these differences and provide more definitive guidance for clinical practice. Because the interventions were evaluated over a short duration and focused on acute postprandial glycemic responses, clinically meaningful changes in body weight were not expected and were therefore not evaluated as a study outcome.

After intervention with the LGI diet alone, a significant reduction in PPG levels was observed when compared to days with the standard Indian diet (Table 4; Figure 1). This finding is consistent with the principles of glycemic control, as consuming foods with a lower GI has been linked to improved postprandial glucose profiles. These observations are in line with previous research that suggests that low-GI diets improve glycemic parameters in contrast to those with carbohydrate-rich meals [24]. Furthermore, a meta-analysis of 54 studies observed that LGI diets significantly improved HbA1c levels among diabetes patients compared to other diets, indicating the strong potential of LGI diets in achieving effective management of T2DM [13]. Therefore, LGI diets may be a potential strategy for achieving better glycemic control in diabetes management in Indian patients.

Limitations

The study's limitations include a small sample size, restricting the generalizability of results. Additionally, the non-availability and cost of CGM devices may hinder their broader use, and some patients might be reluctant to wear such a device for 14 days despite minimal interference with daily activities. Although a two-week run-in period was used to standardize baseline glycemic status and reduce inter-individual variability, the crossover interventions were conducted on consecutive days without a washout interval. Because the interventions evaluated (alpha-glucosidase inhibitors and dietary modifications) primarily exert short-acting, meal-specific effects, significant carryover effects were considered unlikely. Nonetheless, the lack of a washout period constitutes a methodological limitation that must be addressed in subsequent studies featuring extended intervention durations.

## Conclusions

The current study emphasizes the effectiveness of pharmacological interventions (AGIs) and dietary strategies (LGI diets) in achieving rapid postprandial glucose stabilization. These findings support the use of these interventions as valuable tools for managing T2DM, particularly in Indian patients who often consume diets high in carbohydrates, leading to a high glycemic load.
